# Supplementary material for: Current Awareness Status of and Recommendations for Polycystic Ovarian Syndrome: A National Cross-Sectional Investigation of Central Jordan
Source: Int J Environ Res Public Health. 2023 Feb 23;20(5):4018. doi: 10.3390/ijerph20054018 (PMC10001650; doi:10.3390/ijerph20054018)
Supplement: Supplementary file 1 [file ijerph-20-04018-s001.zip › ijerph-2161874-supplementary.pdf]

Supplementary Tables

Supplementary Table S1. Knowledge of PCOS according to field of study

**Item**	**Response**	**Field of Study**			**p-Value**
		**No Education**	**Non-Medical**	**Medical**	
		**n = 161**	**n = 885**	**n = 486**	
In PCOS, there is an increased level of androgen hormones (testosterone)	No	3 (1.9)	36 (4.1)	18 (3.7)	<0.001
	I don't know	117 (72.7)	645 (72.9)	205 (42.2)	
	Yes	41 (25.5)	204 (23.1)	263 (54.1)	
All patients suffering from PCOS have multiple small cysts in their ovaries	No	62 (38.5)	364 (41.4)	174 (35.8)	<0.001
	I don't know	72 (44.7)	296 (33.4)	100 (20.6)	
	Yes	27 (16.8)	225 (25.4)	212 (43.6)	
Obesity may cause PCOS	No	9 (5.6)	63 (7.1)	35 (7.2)	<0.001
	I don't know	56 (34.8)	195 (22.0)	71 (14.6)	
	Yes	96 (59.6)	627 (70.8)	380 (78.2)	
Prediabetes condition (due to decreased insulin action in body) may cause PCOS	No	14 (8.7)	69 (7.8)	42 (8.6)	<0.001
	I don't know	107 (66.5)	450 (50.8)	152 (31.3)	
	Yes	40 (24.8)	366 (41.4)	292 (60.1)	
Irregular or absence of menstrual cycle (period) is a symptom of PCOS	No	10 (6.2)	16 (1.8)	5 (1.0)	<0.001
	I don't know	55 (34.2)	148 (16.7)	30 (6.2)	
	Yes	96 (59.6)	721 (81.5)	451 (92.8)	
Unusual amount of hair growth on different body parts (upper lip, chin, abdomen, breast, ...etc.) is a symptom of PCOS	No	11 (6.8)	37 (4.2)	15 (3.1)	<0.001
	I don't know	63 (39.1)	213 (24.1)	48 (9.9)	
	Yes	87 (54.0)	635 (71.8)	423 (87.0)	
Severe acne problems are a symptom of PCOS	No	20 (12.4)	111 (12.5)	49 (10.1)	<0.001
	I don't know	81 (50.3)	335 (37.9)	113 (23.3)	
	Yes	60 (37.3)	439 (49.6)	324 (66.7)	

Unusual amounts of hair loss from the scalp is a symptom of PCOS	No	21 (13.0)	113 (12.8)	51 (10.5)	<0.001
	I don't know	106 (65.8)	485 (54.8)	204 (42.0)	
	Yes	34 (21.1)	287 (32.4)	231 (47.5)	
PCOS diagnosis can be confirmed through an ultrasound	No	10 (6.2)	59 (6.7)	36 (7.4)	0.001
	I don't know	61 (37.9)	263 (29.7)	106 (21.8)	
	Yes	90 (55.9)	563 (63.6)	344 (70.8)	
Specific blood tests can be used for the diagnosis of PCOS	No	18 (11.2)	80 (9.0)	35 (7.2)	<0.001
	I don't know	87 (54.0)	394 (44.5)	110 (22.6)	
	Yes	56 (34.8)	411 (46.4)	341 (70.2)	
PCOS may lead to diabetes	No	35 (21.7)	152 (17.2)	83 (17.1)	<0.001
	I don't know	102 (63.4)	517 (58.4)	191 (39.3)	
	Yes	24 (14.9)	216 (24.4)	212 (43.6)	
PCOS may lead to weight gain	No	14 (8.7)	58 (6.6)	47 (9.7)	<0.001
	I don't know	67 (41.6)	240 (27.1)	91 (18.7)	
	Yes	80 (49.7)	587 (66.3)	348 (71.6)	
PCOS may lead to pelvic pain	No	2 (1.2)	42 (4.7)	43 (8.8)	<0.001
	I don't know	54 (33.5)	250 (28.2)	108 (22.2)	
	Yes	105 (65.2)	593 (67.0)	335 (68.9)	
PCOS may lead to heart diseases	No	39 (24.2)	203 (22.9)	135 (27.8)	<0.001
	I don't know	111 (68.9)	601 (67.9)	266 (54.7)	
	Yes	11 (6.8)	81 (9.2)	85 (17.5)	
PCOS may lead to hypertension	No	32 (19.9)	159 (18.0)	112 (23.0)	<0.001
	I don't know	107 (66.5)	575 (65.0)	255 (52.5)	
	Yes	22 (13.7)	151 (17.1)	119 (24.5)	
PCOS may lead to infertility (inability to have children)	No	8 (5.0)	92 (10.4)	47 (9.7)	<0.001
	I don't know	57 (35.4)	244 (27.6)	83 (17.1)	
	Yes	96 (59.6)	549 (62.0)	356 (73.3)	
PCOS may lead to pregnancy complications (ex: miscarriage)	No	10 (6.2)	76 (8.6)	38 (7.8)	<0.001

	I don't know	68 (42.2)	329 (37.2)	130 (26.7)	
	Yes	83 (51.6)	480 (54.2)	318 (65.4)	
PCOS may lead to anxiety/ depression/ low self-esteem	No	14 (8.7)	53 (6.0)	11 (2.3)	<0.001
	I don't know	70 (43.5)	275 (31.1)	81 (16.7)	
	Yes	77 (47.8)	557 (62.9)	394 (81.1)	
PCOS may lead to early puberty/ menarche	No	18 (11.2)	131 (14.8)	107 (22.0)	<0.001
	I don't know	118 (73.3)	575 (65.0)	262 (53.9)	
	Yes	25 (15.5)	179 (20.2)	117 (24.1)	
Hormonal therapy may be used to treat PCOS	No	9 (5.6)	25 (2.8)	18 (3.7)	<0.001
	I don't know	88 (54.7)	362 (40.9)	96 (19.8)	
	Yes	64 (39.8)	498 (56.3)	372 (76.5)	
Anti-diabetic medications (ex: Glucophage) may be used to treat PCOS	No	13 (8.1)	33 (3.7)	25 (5.1)	<0.001
	I don't know	90 (55.9)	407 (46.0)	162 (33.3)	
	Yes	58 (36.0)	445 (50.3)	299 (61.5)	
Surgery may be used to remove the ovarian cysts	No	5 (3.1)	84 (9.5)	43 (8.8)	0.001
	I don't know	60 (37.3)	228 (25.8)	109 (22.4)	
	Yes	96 (59.6)	573 (64.7)	334 (68.7)	
Treating PCOS reduces the chance of getting cancer	No	12 (7.5)	85 (9.6)	53 (10.9)	<0.001
	I don't know	100 (62.1)	494 (55.8)	219 (45.1)	
	Yes	49 (30.4)	306 (34.6)	214 (44.0)	
PCOS is an inherited disorder	No	53 (32.9)	280 (31.6)	171 (35.2)	<0.001
	I don't know	92 (57.1)	462 (52.2)	194 (39.9)	
	Yes	16 (9.9)	143 (16.2)	121 (24.9)	

Note: PCOS, polycystic ovary syndrome

Supplementary Table S2. Knowledge of PCOS according to gender

Item	Response	Gender		p-value
		Male	Female	
		n=175	n=1357	
In PCOS, there is an increased level of androgen hormones (testosterone)	No	10 (5.7%)	47 (3.5%)	0.312
	I don't know	106 (60.6%)	861 (63.4%)	
	Yes	59 (33.7%)	449 (33.1%)	
All patients suffering from PCOS have multiple small cysts in their ovaries	No	52 (29.7%)	548 (40.4%)	<0.001
	I don't know	87 (49.7%)	381 (28.1%)	
	Yes	36 (20.6%)	428 (31.5%)	
Obesity may cause PCOS	No	11 (6.3%)	96 (7.1%)	<0.001
	I don't know	77 (44.0%)	245 (18.1%)	
	Yes	87 (49.7%)	1016 (74.9%)	
Prediabetes condition (due to decreased insulin action in body) may cause PCOS	No	13 (7.4%)	112 (8.3%)	<0.001
	I don't know	111 (63.4%)	598 (44.1%)	
	Yes	51 (29.1%)	647 (47.7%)	
Irregular or absence of menstrual cycle (period) is a symptom of PCOS	No	10 (5.7%)	21 (1.5%)	<0.001
	I don't know	72 (41.1%)	161 (11.9%)	
	Yes	93 (53.1%)	1175 (86.6%)	
Unusual amount of hair growth on different body parts (upper lip, chin, abdomen, breast, ...etc.) is a symptom of PCOS	No	11 (6.3%)	52 (3.8%)	<0.001
	I don't know	89 (50.9%)	235 (17.3%)	
	Yes	75 (42.9%)	1070 (78.9%)	
Severe acne problems are a symptom of PCOS	No	25 (14.3%)	155 (11.4%)	<0.001
	I don't know	105 (60.0%)	424 (31.2%)	
	Yes	45 (25.7%)	778 (57.3%)	
Unusual amounts of hair loss from the scalp is a symptom of PCOS	No	15 (8.6%)	170 (12.5%)	<0.001

	I don't know	116 (66.3%)	679 (50.0%)	
	Yes	44 (25.1%)	508 (37.4%)	
PCOS diagnosis can be confirmed through an ultrasound	No	12 (6.9%)	93 (6.9%)	0.001
	I don't know	82 (46.9%)	348 (25.6%)	
	Yes	81 (46.3%)	916 (67.5%)	
Specific blood tests can be used for the diagnosis of PCOS	No	13 (7.4%)	120 (8.8%)	0.001
	I don't know	91 (52.0%)	500 (36.8%)	
	Yes	71 (40.6%)	737 (54.3%)	
PCOS may lead to diabetes	No	26 (14.9%)	244 (18.0%)	0.001
	I don't know	115 (65.7%)	695 (51.2%)	
	Yes	34 (19.4%)	418 (30.8%)	
PCOS may lead to weight gain	No	18 (10.3%)	101 (7.4%)	<0.001
	I don't know	90 (51.4%)	308 (22.7%)	
	Yes	67 (38.3%)	948 (69.9%)	
PCOS may lead to pelvic pain	No	5 (2.9%)	82 (6.0%)	<0.001
	I don't know	84 (48.0%)	328 (24.2%)	
	Yes	86 (49.1%)	947 (69.8%)	
PCOS may lead to heart diseases	No	27 (15.4%)	350 (25.8%)	0.009
	I don't know	128 (73.1%)	850 (62.6%)	
	Yes	20 (11.4%)	157 (11.6%)	
PCOS may lead to hypertension	No	22 (12.6%)	281 (20.7%)	0.038
	I don't know	118 (67.4%)	819 (60.4%)	
	Yes	35 (20.0%)	257 (18.9%)	
PCOS may lead to infertility (inability to have children)	No	6 (3.4%)	141 (10.4%)	<0.001
	I don't know	74 (42.3%)	310 (22.8%)	
	Yes	95 (54.3%)	906 (66.8%)	
PCOS may lead to pregnancy complications (ex: miscarriage)	No	10 (5.7%)	114 (8.4%)	0.001
	I don't know	83 (47.4%)	444 (32.7%)	
	Yes	82 (46.9%)	799 (58.9%)	

PCOS may lead to anxiety/ depression/ low self-esteem	No	6 (3.4%)	72 (5.3%)	<0.001
	I don't know	83 (47.4%)	343 (25.3%)	
	Yes	86 (49.1%)	942 (69.4%)	
PCOS may lead to early puberty/ menarche	No	32 (18.3%)	224 (16.5%)	0.699
	I don't know	110 (62.9%)	845 (62.3%)	
	Yes	33 (18.9%)	288 (21.2%)	
Hormonal therapy may be used to treat PCOS	No	12 (6.9%)	40 (2.9%)	<0.001
	I don't know	95 (54.3%)	451 (33.2%)	
	Yes	68 (38.9%)	866 (63.8%)	
Anti-diabetic medications (ex: Glucophage) may be used to treat PCOS	No	15 (8.6%)	56 (4.1%)	<0.001
	I don't know	110 (62.9%)	549 (40.5%)	
	Yes	50 (28.6%)	752 (55.4%)	
Surgery may be used to remove the ovarian cysts	No	11 (6.3%)	121 (8.9%)	0.001
	I don't know	70 (40.0%)	327 (24.1%)	
	Yes	94 (53.7%)	909 (67.0%)	
Treating PCOS reduces the chance of getting cancer	No	14 (8.0%)	136 (10.0%)	0.052
	I don't know	108 (61.7%)	705 (52.0%)	
	Yes	53 (30.3%)	516 (38.0%)	
PCOS is an inherited disorder	No	33 (18.9%)	471 (34.7%)	<0.001
	I don't know	116 (66.3%)	632 (46.6%)	
	Yes	26 (14.9%)	254 (18.7%)	
